# Prevalence and natural history of variants in the *ANKRD26* gene: a short review and update of reported cases

**DOI:** 10.1080/09537104.2022.2071853

**Published:** 2022-05-19

**Authors:** Hrushikesh Vyas, Ahmad Alcheikh, Gillian Lowe, William S Stevenson, Neil V Morgan, David J Rabbolini

**Affiliations:** 1 Institute of Cardiovascular Sciences, College of Medical and Dental Sciences, University of Birmingham, Birmingham, UK; 2 Northern Blood Research Centre, Kolling Institute, University of Sydney, Sydney, Australia; 3 Comprehensive Care Haemophilia Centre, University Hospital Birmingham NHS Foundation Trust, Birmingham, UK; 4 Department of Haematology and Transfusion Medicine, Royal North Shore Hospital, Sydney, Australia

**Keywords:** *ANKRD26*, inherited thrombocytopenia AML, MDS, platelet disorder, thrombocytopenia

## Abstract

*ANKRD26* is a highly conserved gene located on chromosome 10p12.1 which has shown to play a role in normal megakaryocyte differentiation. *ANKRD26*-related thrombocytopenia, or thrombocytopenia 2, is an inherited thrombocytopenia with mild bleeding diathesis resulting from point mutations the 5ʹUTR of the *ANKRD26* gene. Point mutations in the 5ʹUTR region have been shown to prevent transcription factor-mediated downregulation of *ANKRD26* in normal megakaryocyte differentiation. Patients with *ANKRD26*-related thrombocytopenia have a predisposition to developing hematological malignancies, with acute myeloid leukemia and myelodysplastic syndrome most commonly described in the literature. We review the clinical features and biological mechanisms of *ANKRD26*-related thrombocytopenia and summarize known cases in the literature.

## Introduction

Inherited thrombocytopenias (IT) are a group of disorders which present with a reduced platelet count but varied functional and morphological platelet characteristics. Some ITs are associated with extra-hematological manifestations such as sensorineural deafness (*MYH9*-related disease, *DIAPH1*-related disease) or myopathy (Storkmorken syndrome) whilst others have a predisposition to hematological malignancies such as acute myeloid leukemia (AML), acute lymphoblastic leukemia (ALL), chronic myeloid leukemia (CML), and myelodysplastic syndrome (MDS) [1–4]. At least 40 genes and their mutations have been implicated in the development of inherited thrombocytopenia [3,5,6]. Definitive identification of the genetic nature of thrombocytopenia can be important as some forms differ in disease history and prognosis. Molecular diagnosis may impact clinical management, including monitoring for associated hematological malignancies.

*ANKRD26*-related thrombocytopenia (*ANKRD26*-RT), also known as thrombocytopenia 2 (THC2) (OMIM #188000), is a non-syndromic autosomal dominant thrombocytopenic disorder [7]. Though first described in a large Italian family, in which 17 individuals were affected and the gene locus on chromosome 10 identified using linkage analysis and candidate mutation screening, localization of pathological variants to the 5’ untranslated region (5ʹUTR) of *ANKRD26* was not made until 2011 [8,9]. Since then, multiple causative variants have been shown to be the result of single nucleotide changes in the highly conserved 5’ UTR region of the gene [10,11]. Case reports of variants in the coding region segregating with thrombocytopenia have also been reported [12–14].

## Clinical features

Patients with *ANKRD26*-RT typically have lifelong mild (100–150 x10^9^ cells/L) to moderate (50–99 x10^9^ cells/L) thrombocytopenia, although counts may temporarily normalize in response to infection or inflammation [5,15]. The bleeding phenotype is variable. Most have a normal or mild bleeding phenotype without a history of spontaneous or prolonged surgical bleeding [7,16]. However, some individuals experiencing spontaneous epistaxis, bruising, or menorrhagia have also been reported. Morphologically, platelets appear normal in size and mean platelet volume is usually within normal range. Under light microscopy, platelets appear predominantly normogranular, with occasional hypogranular forms noted in some individuals. By electron microscopy, a reduction in alpha-granules has been described, as well as, increased particulate proteasome-rich cytoplasmic structures, the cause of which is yet to be clarified [16,17]. Platelet aggregation studies are often normal, however, reduced platelet responses to arachidonic acid and epinephrine have been reported. GPIa is commonly reduced when evaluated by flow cytometry and up to a sevenfold increase in serum thrombopoietin levels is seen in some cases [10,11,16]. Dysmegakaryopoiesis with an increase in small and hypolobulated megakaryocytes with reduced cytoplasmic volume is commonly cited in those who have undergone bone marrow biopsies [11,18]. Hemoglobin and white cell counts are generally within the normal range, with inconsistent reports of leukocytosis and erythrocytosis described [10,19].

## Predisposition to malignancy

A clinically important feature of *ANKRD26*-RT reported in the literature is the increased risk of developing hematological malignancy. AML and MDS are the most commonly described, though there are some reports of lymphoid malignancies and a single case report of a patient with a 5ʹUTR mutation developing multiple myeloma [6,10–12,16,20]. An extended case series of 118 subjects with confirmed or highly probable *ANKRD26*-RT revealed an 8% incidence of myeloid malignancy (AML, MDS, and chronic myeloid leukemia (CML)) [10]. An estimated 24-fold increase in acute leukemia incidence alone is reported compared to the general population in this study, and there have been multiple other case reports describing affected patients or their relatives developing hematological malignancy (Table I).

**Table I.** Malignancies associated with variants in the 5ʹUTR sequence of *ANKRD26.*

Reference	Individual families by mutation	Number of affected patients in described families with a confirmed diagnosis of ANKRD26-RT with personal or family history of malignancy	Described malignancy developing in a participant with confirmed ANKRD26-RT	Malignancy described in a 1^st^ degree relative of the screened participant with ANKRD26-RT where the affected relative was unavailable to provide samples to confirm a diagnosis of ANKRD26-RT
[11], [21]	c.-118 C > A	N/A	-	Leukemia (undefined)
	c.-125 T > G	7	Acute Leukemia (5 x myeloid, 2x undefined)	-
	c.-125 T > G			-
	c.-127 A > T			Leukemia (undefined)
	c.-127 A > T			-
	c.-134 G > A			-
	c.-128 G > A	2	CML, MDS and CLL (MDS and CLL diagnosed in same patient)	-
	c.-127 A > T	1	CLL	-
[12]	c.-125 T > G	1	AML	-
[19]	c.-128 G > A	6	2 x AML	-
	c.-127 A > T	5	1x AML	-
	c.-127 A > T	4	MDS	-
	c.134 G > C	4	CML	-
[20]	c.-128 G > A	1	Multiple myeloma	-
[22]	c.-127 A > T	1	CML	N/A
[23]	c.-116 C > T	1	CMML	-
[24]	c.-140 C > G	1	-	Prostate Ca
	c.-140 C > G	1	Renal Ca	-
	c.-128 G > A	1	-	AML
	c.-140 C > G	1	Breast Ca	-
	c.-140 C > G	1	AML	-
[25]	c.-118 C > T	2	MDS/AML (2 individuals with MDS/AML)	1 additional relatives with MDS/AML
[26]	c.-118 C > T	1	-	Leukemia (undefined)
[27]	c.-118 C > T	1	-	Leukemia (undefined)

## Differential diagnosis

Diagnosis of *ANKRD26*-RT may be difficult due to the lack of distinct clinical and laboratory characteristics [16]. In many cases, patients may be misdiagnosed with immune thrombocytopenia (ITP), which should be a diagnosis of exclusion. This is especially the case if there is no obvious family history of thrombocytopenia or if the patient has had fluctuating platelet counts in the past [28,29]. There are also documented cases of patients being incorrectly diagnosed with MDS on the basis of persistently low platelet counts and bone marrow biopsy demonstrating dysmegakaryopoiesis, both of which may be features of *ANKRD26*-RT [30].

Other ITs to consider which may also present with thrombocytopenia with normal platelet size include *RUNX1*-related thrombocytopenia (*RUNX1*-RT), *ETV6*-related thrombocytopenia (*ETV6*-RT) and *CYCS*-related thrombocytopenia (*CYCS*-RT) [3]. Of these, *RUNX1*-RT demonstrates a 30–40% lifetime risk of developing MDS/AML and *ETV6*-RT conferring a 20% lifetime risk of B-ALL with a 30% overall lifetime risk of hematological malignancy [1,5,31–33].

## Pathophysiology

### Regulation of *ANKRD26* expression

**Figure 1.**
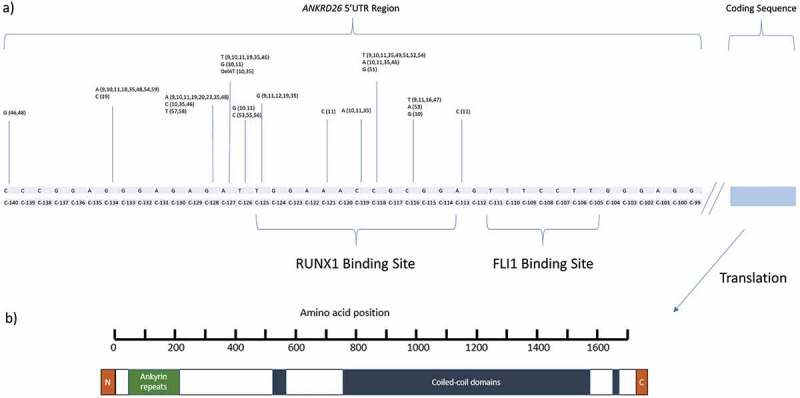
(a) Schematic structure of 5ʹUTR of *ANKRD26* mapping single point variants currently identified in the literature. (b) General structure of ANKRD26 protein [36].

*ANKRD26* is located on chromosome 10p12.1 and contains 34 exons that result in a number of protein isoforms expressed at low levels in multiple human tissues, including platelets, leukocytes, adrenal glands, prostate, ovary, liver, spleen, and central nervous system [34,35]. *ANKRD26* shares regions of homology with the POTE family of genes that are characterized by ankyrin repeats (involved in protein–protein interactions) close to the N-terminal region and a helical region that forms coiled-coil domains similar to that of spectrins, suggesting involvement in signal transmission across the plasma membrane (Figure 1b) [36–38]. *ANKRD26* is highly conserved between the different species, suggesting an important function [38,39]. Mouse Ankrd26 protein localizes to the cell membrane in cell lines and human ANKRD26 protein is identified in centriolar distal appendages and cilial basal bodies in human cell lines [39–41].

In healthy subjects, *ANKRD26* mRNA expression is high in CD34+ progenitor cells and immature megakaryocytes, and decreases over time, becoming almost undetectable in mature platelets [42]. Predictive software identified two transcription factors that bind the *ANKRD26* 5’ UTR: runt-related transcription factor 1 (RUNX1) and friend leukemia integration 1 (FLI1). Knockdown of either gene in human megakaryocytes led to increased *ANKRD26* mRNA expression, while overexpression of these genes in K562 cell lines led to decreased *ANKRD26* mRNA. The effect was synergistic when both genes were overexpressed. Other studies suggest metabolic and/or inflammatory factors may also influence mRNA expression in mouse adipose tissue and human leukocytes through epigenetic alterations [43,44]. Taken together, these lines of evidence suggest expression of *ANKRD26* is regulated by interaction of transcription factors with the 5ʹUTR and epigenetic alterations.

### Mechanisms contributing to thrombocytopenia

The 5ʹUTR regulatory sequence of *ANKRD26* is the most common site of single nucleotide variants identified in *ANKRD26*-RT. A total of 318 patients reported in the literature have a single point variant in this region (Table II). The most common variants include c.-128 G > A, c.-134 G > A and c.-127A>T, and lie close to RUNX1 and FLI1 binding sites (Figure 1a). These single nucleotide variants in the 5’ UTR alter *ANKRD26* transcription by preventing RUNX1/FLI1-mediated repression and result in persistently high levels of *ANKRD26* mRNA at all stages of platelet development [42,52]. Electron microscopy of megakaryocyte cell lines from these patients showed slightly lower ploidy, decreased granule concentration, and abnormal proplatelet formation compared to controls.

**Table II.** A summary of point mutations in the 5’ untranslated region of the *ANKRD26* gene as reported in the literature. Mean averages reported where data available and ranges and number of individuals accounted for are in brackets.

5’ UTR mutation	Number of families	Number of patients	Mean platelet count (x10^9/L)	Mean MPV (fl)	Mean Hb (g/L)	Mean WBC (x10^9/L)	Mean TPO (pg/mL)	Reference
c.-113A>C	1	3	85 (23–176) (n = 3)	11.43 (11–12.1) (n = 3)	12.53 (11.9–13.2) (n = 3)	3.9 (2.71–5.6) (n = 3)	-	[11]
c.-116C>A	1	1	82 (n = 1)	9.3 (n = 1)	13.5 (n = 1)	11.9 (n = 1)	-	[45]
c.-116C>G	1	3	70.6 (45–107) (n = 3)	9.55 (9.4–9.7) (n = 3)	12.85 (12.1–13.6) (n = 3)	11.7 (10.6–12.8) (n = 3)	-	[10]
c.-116C>T	3	10	52.14 (36–74) (n = 7)	10.85 (10.5–11.2) (n = 2)	14.95 (13.9–16) (n = 2)	11.04 (6.08–16) (n = 2)	-	[9,11,16,23]
c.-118C>A	4	10	58.2 (15–100) (n = 10)	9.59 (8.68–10.5) (n = 10)	15.08 (12.3–16.3) (n = 10)	9 (6.8–12.6) (n = 6)	206 (132–280) (n = 2)	[10,11,22,42]
c.-118C>T	10	16	47.69 (7–73) (n = 13)	9.84 (7.7–11.6) (n = 12)	13.1 (10.4–15.3) (n = 10)	9.88 (5.61–17.5) (n = 7)	258.3 (39–497) (n = 4)	[9–11,25–27,42,46]
c.-118C>G	1	1	43 (n = 1)	11 (n = 1)	-	-	-	[26]
c.-119C>A	3	5	50.96 (36–81) (n = 5)	9.3 (6.2–11.3) (n = 5)	13.5 (12.2–14.2) (n = 5)	7 (4.7–9.6) (n = 4)	-	[10,11,42]
c.-121A>C	1	3	63 (28–87) (n = 3)	10.26 (10–10.8) (n = 3)	15.6 (12.8–17) (n = 3)	15.45 (11.6–21.33) (n = 3)	-	[11]
c.-125 T > G	6	12	28.25 (7–12) (n = 4)	7.98 (6.5–10.8) (n = 4)	15.45 (12–17.3) (n = 4)	10.45 (7–13.8) (n = 4)	190.2 (148.4–232) (n = 2)	[9,11,12,19,42]
c.-126 T > C	3	3	22.3 (19–27) (n = 3)	12.2 (n = 1)	14.35 (13.8–14.9) (n = 2)	5.5 (n = 1)	-	[45,47,48]
c.-126 T > G	2	4	36.74 (14–80) (n = 4)	9.98 (8.1–10.9) (n = 4)	15.67 (13.7–16.5) (n = 4)	8.88 (7.3–12.19) (n = 4)	-	[10,11]
c.-127A>G	3	13	93.29 (46–147) (n = 13)	8.39 (6.48–9.5) (n = 13)	15.3 (12.4–18.3) (n = 11)	8.69 (6.1–11.7) (n = 13)	-	[10,11]
c.-127A>T	14	54	44.78 (10–94) (n = 34)	9.98 (7.1–14.3) (n = 30)	14.12 (10.5–17.2) (n = 31)	8.71 (5.3–12.1) (n = 23)	165.5 (106–190) (n = 8)	[9–11,19,22,42]
c.-127delAT	2	10	51.86 (26–96) (n = 10)	10.21 (8.7–11) (n = 10)	14.28 (10.4–16.6) (n = 10)	7.96 (5.8–13.4) (n = 10)	140.25 (97–178) (n = 4)	[10,42]
c.-128 G > A	24	76	32.4 (5–75) (n = 51)	8.77 (6.3–11.6) (n = 31)	14.58 (10.2–18.4) (n = 49)	9.18 (5.1–21) (n = 44)	136.67 (104–191) (n = 3)	[9–11,19,20,24,30,42]
c.-128 G > C	2	4	38.75 (24–81) (n = 4)	9.95 (7.9–14) (n = 4)	13.48 (12–14.9) (n = 4)	9.35 (6.6–12.1) (n = 2)	185 (n = 1)	[10,22,42]
c.-128 G > T	2	13	36.92 (19–70) (n = 13)	9.73 (7.9–12.5) (n = 7)	12 (6.9–14.9) (n = 13)	9.64 (4.98–14.4) (n = 13)	163 (49.76–288.58) (n = 7)	[49,50]
c.-134 G > A	15	59	44.89 (7–106) (n = 45)	8.69 (5.7–11.1) (n = 44)	14.76 (10.5–17.8) (n = 43)	9.34 (5.10–16.4) (n = 31)	-	[9–11,18,24,42,46,51]
c.-134 G > C	2	7	-	-	-	-	-	[19]
c.-134 G > A and c.-140c>G	1	1	70 (n = 1)	9 (n = 1)	12 (n = 1)	-	-	[24]
c.-140C>G	6	9	91.78 (50–200) (n = 9)	9.56 (8–11.7) (n = 9)	12.73 (9.5–14.1) (n = 9)	-	-	[22,24]
Totals:	107	317						

Components of the mitogen-activated protein kinase (MAPK) pathways appear to play an important role in this process, because the megakaryocytic changes were associated with increased ERK phosphorylation, and were abrogated by *ANKRD26* knockdown or ERK pathway inhibition using a MEK inhibitor [42]. MEK (also known as MKK or MAP2K) inhibition has previously been shown to increase ploidy and proplatelet formation in thrombopoietin-stimulated human megakaryocytes [53]. Therefore, the evidence suggests that persistence of ANKRD26 expression in *ANKRD26*-RT leads to persistent ERK activation, which may in turn be responsible for, or contribute to, reduced megakaryocyte ploidy, impaired proplatelet formation, and subsequent thrombocytopenia.

At least one other mechanism for increased *ANKRD26* expression has been described in a thrombocytopenic family using long-read genomic sequencing. In this pedigree, a complex structural variant resulting in a paired duplication-inversion of part of the *ANKRD26* gene was identified that caused a juxtaposition of the promotor of WAC and exons 10–34 of *ANKRD26*. This gene duplication resulted in high WAC-*ANKRD26* mRNA levels and increased ERK phosphorylation similar to the phenotype caused by 5ʹUTR variants [54].

To date, no animal model of *ANKRD26*-RT has been described. A mouse *Ankrd26* knockdown model did not report blood abnormalities but showed mice with hyperphagia, organomegaly, obesity, and reduced expression of ciliary proteins in the brain [40,43,55].

### ANKRD26 function in other cellular processes - the centrosome

As suggested by its localization, ANKRD26 appears to play a role in centriole biology [41]. Centrioles are important in ciliogenesis and motility. They are components of centrosomes that have been implicated in cancer pathogenesis [56,57]. Centrosome amplification triggers p53-dependent apoptosis through activation of a multiprotein complex known as the PIDDosome [56]. In centrosome amplification (e.g. cytokinesis failure), ANKRD26 recruits the p53-induced death domain protein 1 (PIDD1) to the centriole distal appendages to form part of the PIDDosome [57–59]. When ANKRD26 is inactivated, cells cannot sustain PIDDosome assembly and show enhanced growth following centrosome amplification [57,59]. Whether these actions of ANKRD26 protein play any role in the pathogenesis of *ANKRD26*-RT is unknown.

### Concluding remarks

*ANKRD26*-RT is characterized by a relatively nonspecific phenotype of mild to moderate thrombocytopenia with normal platelet size and function. Most individuals lack significant mucocutaneous bleeding symptoms. A concerning association with hematological malignancy has been observed in cohorts with variants in the 5ʹUTR region. However, precise prevalence estimates and strategies to guide clinical monitoring, counseling, and treatment will only be possible through further analysis of large patient cohorts and exploration of the pathophysiological mechanisms underpinning this disorder.
